# The Great Migration and the Urban-Rural Divide: Lonely Life Expectancy in China

**DOI:** 10.1093/geroni/igab046.139

**Published:** 2021-12-17

**Authors:** Xueqing Wang, James Raymo

**Affiliations:** Princeton University, Princeton University, New Jersey, United States

## Abstract

After decades of below replacement fertility, China is now experiencing rapid population aging and the lives of the growing older population are being shaped by massive social and economic change. Of particular importance, is the large-scale migration of working-age adults from rural areas to large cities in search of job opportunities. The departure of migrants from their rural hometowns has resulted in a large population of left-behind older men and women. This distinctively Chinese demographic phenomenon has spurred scholarly interest in the emotional well-being of this older left-behind population, but careful demographic description of aging, migration, grandparenting, and loneliness has yet to be conducted. We bridge this gap by describing the average remaining life spent lonely by older men and women in China. We use Sullivan’s method to calculate lonely life expectancy by urban/rural residence and by the migration status of adult children (as proxied by the presence or absence of coresiding children). We use data from the Harmonized version of the Chinese Health and Retirement Longitudinal Study and focus the analysis on adults aged 55-100. Preliminary results show that, at age 55, women on average spend 9% more of remaining life lonely than men and that rural men and women spend more of their remaining life lonely than their urban counterparts. We will extend these life table analyses by conducting multivariate analyses of the correlates of loneliness in urban and rural China to better understand the role of migration and grandparenting responsibilities.

